# Primary Localized Cutaneous Amyloidosis Presenting With Tumor-Like Swelling in the Lower Extremity: A Rare Presentation

**DOI:** 10.7759/cureus.82228

**Published:** 2025-04-14

**Authors:** Ahmed Almukhlifi, Ayman Ibrahim, Ahmed Hazazzi, Noorah Almadani

**Affiliations:** 1 Department of Medicine, Ministry of National Guard Health Affairs, Riyadh, SAU; 2 Department of Hematology, King Abdulaziz Medical City, Riyadh, SAU; 3 Department of Internal Medicine, Ministry of National Guard, Riyadh, SAU; 4 Department of Pathology and Laboratory Medicine, King Abdulaziz Medical City, Riyadh, SAU

**Keywords:** al kappa-type amyloidosis, amyloidoma, amyloidosis, extremity, nodular amyloidosis, primary localized cutaneous amyloidosis, soft tissue swelling, tumor

## Abstract

Amyloidosis consists of a spectrum of disease ranging from primary localized forms to secondary forms due to systemic disease. Amyloidoma, a variant of localized amyloidosis, is the least frequent manifestation of tissue amyloid deposition, which can be either amyloid A (AA) or amyloid light-chain (AL) type. Soft tissue amyloidoma is uncommon and mostly affects the abdomen and mediastinum and rarely affects the extremities. We present a middle-aged woman who developed a tumor-like swelling in the extremity, in which comprehensive investigations revealed features of primary amyloidosis with no evidence of internal involvement, representing a rare presentation.

## Introduction

Amyloidosis is caused by the buildup of amyloid protein in different tissues. It is classified clinically into four types: cutaneous amyloidosis, hemodialysis-associated amyloidosis, familial amyloidosis, and systemic amyloidosis. Unlike systemic amyloidosis, primary cutaneous amyloidosis is confined to the skin and does not affect other organs. While about 10% of instances of primary localized cutaneous amyloidosis (PLCA) may be genetic, the majority of cases are sporadic [1]. PLCA is divided into three subtypes: nodular, lichenoid, and macular amyloidosis. Primary cutaneous nodular amyloidosis (PCNA) is the least common subtype [2]. A primary amyloidoma is described as a single mass of amyloid protein that lacks signs of systemic amyloidosis. Amyloidomas appear as non-tender, hard lumps distributed throughout the body. They manifest on the skin as single, irregular, brown to erythematous plaques [3]. Early detection of cutaneous amyloidosis and assessment for systemic involvement are crucial. It is extremely uncommon for this subtype of amyloidomas to affect the soft tissue of the extremities. It differs from its equivalents at other anatomic sites in several respects, and its pathophysiology is not well known. Few case reports described a similar presentation with no systemic involvement. We describe a recent instance of localized soft tissue amyloidoma of the extremities. Our goal is to add information to a scarce literature about this uncommon entity.

## Case presentation

**Figure 1:**
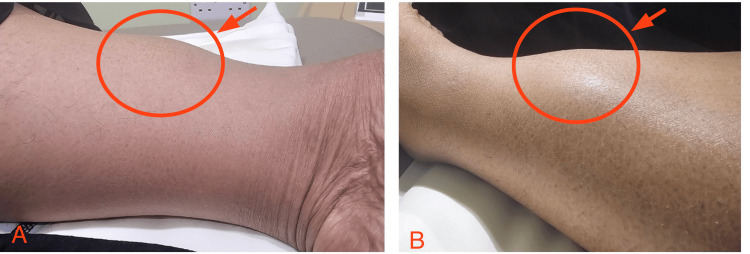
Swelling of the left leg Tumor-like swelling overlying the distal left tibia with smooth surface and no skin changes (arrowheads). (A) Medial view. (B) Lateral view.

A 48-year-old woman with a history of hypertension, diabetes mellitus, and dyslipidemia, who is functionally independent and ambulatory, has been following up with the orthopedic clinic for left knee pain. The pain started gradually over the last couple of weeks and was accompanied by a mass that appeared three years ago, leading to knee pain while walking. Recently, the mass has gradually increased in size. It is soft, round, painless, and located at the distal anterior shaft of the left tibia, measuring 4 × 4 cm (Figure 1).

**Figure 2:**
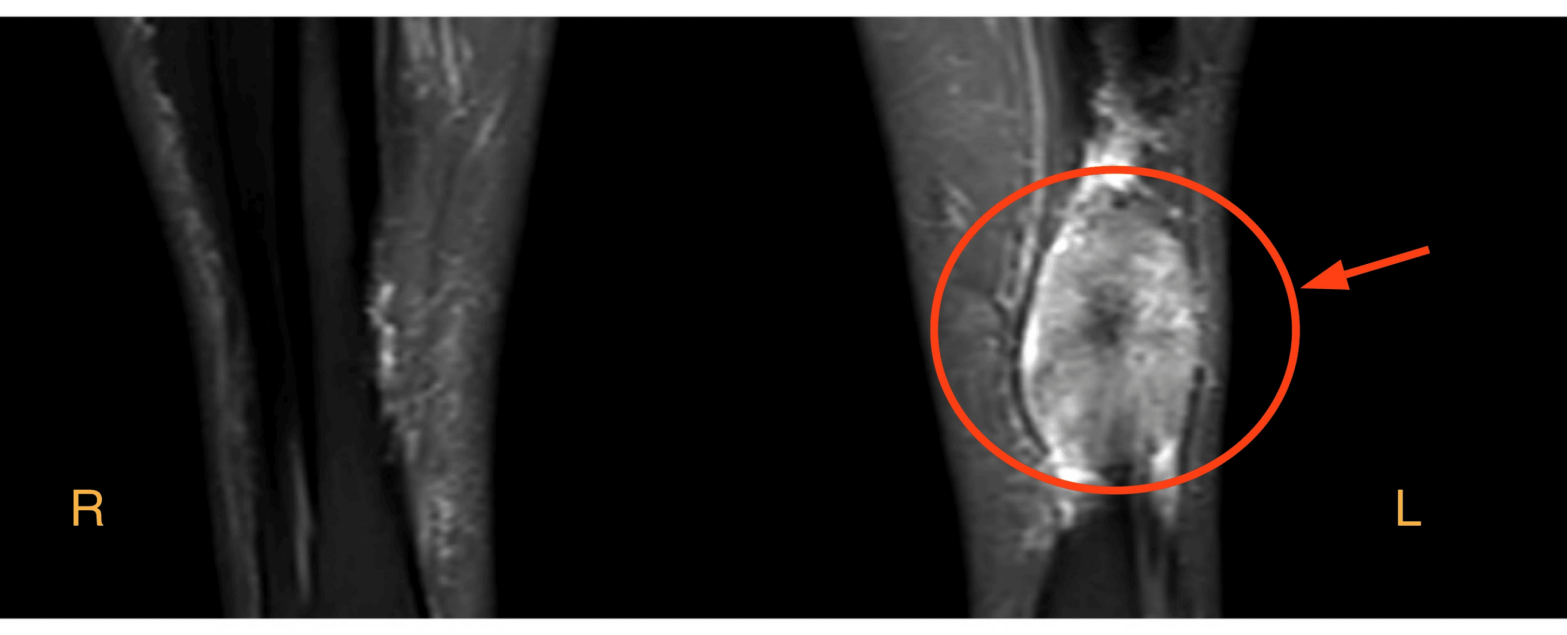
MRI of the legs There is a distal tibial well-demarcated soft tissue mass overlying the anteromedial tibia (arrowhead) associated with spiculated periosteal reaction (likely a superficial invasion). The mass contains a few internal calcification and demonstrates heterogeneous T1WI isointensity and heterogeneous T2 hyperintensity with heterogeneous postcontrast enhancement measuring 6.3 × 4.1 × 1.3 cm worrisome of underlying soft tissue sarcoma. T1WI: T1-weighted imaging; R: right; L: left

On examination, there were no skin changes, tenderness, pruritus, or neurovascular compromise, and a full range of motion was maintained. There was no previous history of trauma, B symptoms, or malignancy in the family. MRI of the left leg revealed a heterogeneously enhancing soft tissue mass concerning for sarcoma (Figure 2). Tissue biopsy was obtained, the histopathological appearance was compatible with amyloidosis, and the patient was referred later to hematology.

**Figure 3:**
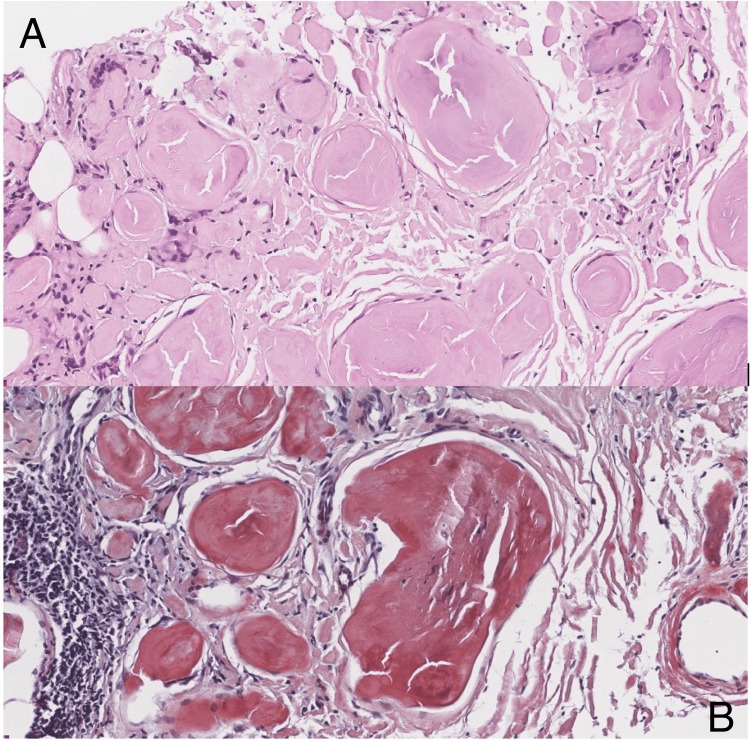
Histopathological features of amyloidosis Histology (hematoxylin and eosin stain, 400×) reveals that homogeneous material is present as extracellular deposits and displays characteristic cracks. These densely eosinophilic material separated by connective tissue with chronic inflammation (A). Same section stained with Congo red (400×). The amyloid deposits have a bright orange-red appearance (B).

In the hematology clinic, laboratory tests, including a complete blood count, a complete metabolic panel, serum protein electrophoresis, and immunofixation of serum immunoglobulin light chains, were all normal. A bone marrow biopsy was negative for multiple myeloma, showing only 1% plasma cells with no morphologic or immunophenotypic evidence of plasma cell myeloma. Echocardiography was normal, and tests for hepatitis B and C and HIV were negative. Additionally, positron emission tomography (PET) was negative for internal visceral involvement (Table 1). The tissue sample was sent to the Mayo Clinic laboratory for analysis. Liquid chromatography with tandem mass spectrometry (LC-MS/MS) was performed on peptides extracted from Congo red-positive microdissected areas of the paraffin-embedded specimen, which detected a peptide profile consistent with amyloid light-chain (AL) kappa-type amyloidosis deposits (Figure 3).

**Table 1:** Laboratory investigations TSH: thyroid-stimulating hormone; Ig: immunoglobulin; HIV: human immunodeficiency virus; GFR: glomerular filtration rate

Exam	Result	Reference range
TSH	2.1	0.35-4.9 mIU/l
Hemoglobin	127	120-160 g/l
White blood cell count	7.8	4-11 * 10^9^/l
Platelets	316	150-400 * 10^9^/l
Alpha 2	10.2	5.1-8.5 g/l
Beta 1	4.6	3.4-5.2 g/l
Beta 2	4.9	2.3-4.7 g/l
Gamma	15	2.3-4.7 g/l
Alpha 1	3.2	2.1-3.5 g/l
Serum immunofixation	Absent (no monoclonal bands)	Absent
M-spike	0%	0%
IgA	2.9	0.84-4.9 g/l
IgM	0.85	0.35-2.4 g/l
IgG	16	6-16 g/l
Free kappa	43.3	3.3-19.4 mg/l
Free lambda	19.4	5.7-26.3 mg/l
Kappa/lambda ratio	2.2	0.26-1.65 ratio
Hepatitis B and C	Nonreactive	Nonreactive
HIV	Nonreactive	Nonreactive
Creatinine	46	44-97 umol/l
GFR	134	~60 ml/min/1.73 m^2^

## Discussion

Amyloidosis is a well-known consequence of malignant neoplasms, plasmacytic dyscrasias, hemodialysis, and chronic inflammation. It can be classified as either localized or systemic based on the patterns of deposits. There are three forms of PLCA: lichenoid, macular, and nodular [4]. Lichenoid amyloidosis is characterized by hyperpigmented papules on the shins, while macular amyloidosis is characterized by tiny, rippling, hyperpigmented macules on the upper back. Pruritus is linked to both of these conditions. Skin-colored nodules ranging in size from several millimeters to several centimeters are a hallmark of nodular amyloid, which is extremely uncommon. The AL type of amyloid constitutes the amyloid in nodular amyloidosis, comparable to that in primary systemic amyloidosis. According to several studies, there is a 7-50% chance that nodular amyloidosis will progress to systemic amyloidosis [5,6]. For this reason, assessing patients for systemic amyloidosis is crucial. In our case, systemic amyloidosis was ruled out by laboratory tests and bone marrow biopsy. Additionally, the patient has no significant background of malignancy or autoimmune disease. Furthermore, the PET scan was negative for internal visceral involvement.

Amyloidoma, or localized amyloidosis with a tumor-like appearance, is uncommon and typically linked to immunocytic dyscrasias [7]. Amyloidomas are primarily seen in visceral organs such as the breast, urinary tract, gastrointestinal tract, and lung. In our instance, soft tissue amyloidomas are quite rare, and there are discrepancies regarding their actual locations in soft tissues due to inconsistent criteria and terminology. Only five cases from the largest known series are cited by the authors, who detail 14 cases. However, only one of their 14 cases occurred in the soft tissues of the right flank; the other 13 were in the retroperitoneum, mediastinum, and mesentery [7-10]. Unlike systemic amyloidosis, the cause of amyloidoma production is unknown, and no studies have been undertaken on this unusual condition. Current theories imply that amyloidomas are caused by the localized synthesis of monoclonal light chains that aggregate, rather than circulating misfolded proteins resulting in deposits [11]. 

Nodular amyloidosis is frequently accompanied by subcutaneous tissue involvement. Clinically, it presents as one or more waxy papules or nodules that affect the genital area, head/neck, or legs. Amyloidoma shares features with the nodular variant, as it has deep tissue involvement and AL-type amyloid deposits. There is a scarcity of data on PCLA presenting with soft tissue swelling or a mass-like tumor. Most of the cases in the literature have skin changes like bruises, plaques, macules, or nodules. Two case reports showed similar patient presentations with a mass measuring 2-3 cm in the lower extremities [12,13]. No definitive guidelines on the best treatment options for PLCA can be established based on the current literature. Options include surgical excision, cryotherapy, and phototherapy. Patients should be advised that therapy options available are based on case reports and expert judgment, rather than clinical trials [14]. Additionally, management should be tailored to the patient's symptoms and preferences; in our patient's case, she preferred no intervention and to continue follow-up.

## Conclusions

Amyloidoma is a rare pathological entity and extremely uncommon in the soft tissue of the extremity. This case highlights the importance of early identification of amyloidoma and the need to exclude systemic disease. Further research is warranted to enhance the understanding of its pathogenesis and to establish more effective management strategies.
